# Analysis of the anatomic eligibility for transcarotid artery revascularization in Chinese patients who underwent carotid endarterectomy and transfemoral carotid artery stenting

**DOI:** 10.3389/fcvm.2022.1045598

**Published:** 2023-01-06

**Authors:** Weijian Fan, Weihao Shi, Shuangshuang Lu, Wencheng Guo, Jindong Tong, Jinyun Tan, Bo Yu

**Affiliations:** ^1^Department of Vascular Surgery, Shanghai Pudong Hospital, Fudan University Pudong Medical Center, Shanghai, China; ^2^Fudan Zhangjiang Institute, Shanghai, China; ^3^Department of Vascular Surgery, Huashan Hospital of Fudan University, Shanghai, China; ^4^Department of Nursing, Huashan Hospital of Fudan University, Shanghai, China

**Keywords:** carotid artery stenosis, transcarotid artery revascularization, anatomic eligibility, carotid endarterectomy, transfemoral carotid artery stenting, circle of Willis, clinical outcomes

## Abstract

**Objective:**

Transcarotid artery revascularization (TCAR) is thought to be a promising technique and instrument for treating carotid stenosis with favorable outcomes. Since there remain several differences in anatomic characteristics among races, this study was conducted to investigate the anatomic eligibility of TCAR in Chinese patients who underwent carotid revascularization.

**Methods:**

A retrospective review of patients with carotid stenosis from 2019 to 2021 was conducted. The anatomic eligibility of TCAR was based on the instruction of the ENROUTE Transcarotid Neuroprotection System. The carotid artery characteristics and configuration of the circle of Willis (CoW) were evaluated by CT angiography. The demographic and clinical characteristics and procedure-related complications were recorded. Logistic regression was used to analyze the independent factors for TCAR eligibility.

**Results:**

Of 289 consecutive patients [222 for carotid endarterectomy (CEA) and 67 for transfemoral carotid artery stenting (TF-CAS)] identified, a total of 215 patients (74.4%) met TCAR anatomic eligibility. Specifically, 83.7% had mild common carotid artery (CCA) puncture site plaque, 95.2% had 4–9 mm internal carotid artery diameters, 95.8% had >6 mm CCA diameter, and 98.3% had >5 cm clavicle to carotid bifurcation distance. Those who were female (OR, 5.967; 95% CI: 2.545–13.987; *P* < 0.001), were of an older age (OR, 1.226; 95% CI: 1.157–1.299; *P* < 0.001), and higher body mass index (OR, 1.462; 95% CI: 1.260–1.697; *P* < 0.001) were prone to be associated with TCAR ineligibility. In addition, 71 patients with TCAR eligibility (33.0%) were found to combine with incomplete CoW. A high risk for CEA was found in 29 patients (17.3%) with TCAR eligibility, and a high risk for TF-CAS was noted in nine patients (19.1%) with TCAR eligibility. Overall, cranial nerve injury (CNI) was found in 22 patients after CEA, while 19 of them (11.3%) met TCAR eligibility.

**Conclusion:**

A significant proportion of Chinese patients meet the anatomic criteria of TCAR, making TCAR a feasible treatment option in China. Anatomic and some demographic factors play key roles in TCAR eligibility. Further analysis indicates a potential reduction of procedure-related complications in patients with high-risk carotid stenosis under the TCAR procedure.

## Introduction

According to the results from large randomized trials, carotid endarterectomy (CEA) and transfemoral carotid artery stenting (TF-CAS) have been proven as safe and effective treatments of choice for patients with carotid stenosis (1–4). However, some typical anatomical and physiological characteristics are relatively risky for both CEA and TF-CAS, with a higher incidence of perioperative complications. Transcarotid artery revascularization (TCAR) recently emerged as an alternative to CEA and TF-CAS, showing promising results in carotid revascularization (5–7). The temporary shunt is inserted between the common carotid artery (CCA) and femoral vein through the ENROUTE Transcarotid Neuroprotection System (Silk Road Medical Inc, Sunnyvale, California) to initiate dynamic flow reversal for preventing distal embolization, and stent implantation is completed similar to the TF-CAS procedure (8). Of note, the safety and efficacy of TCAR are supported by novel but promising results. A total of 45 studies with 14,588 patients who underwent TCAR for carotid artery stenosis were retrospectively analyzed. Results showed that the overall peri-procedural all-cause mortality and stroke rate were 0.5 and 1.3%, respectively (9). Hence, in order to test the applicability of TCAR to the general population, several studies assessed the percentage of carotid arteries that met anatomic criteria for TCAR (10, 11). Even though most of the candidates in these studies were white people, the studies are still useful in indicating the significant differences between eligible and ineligible groups for TCAR.

The Vascular Quality Initiative (VQI) only represents different practice patterns in America, therefore verification of TCAR in different nations and with different races is needed to ensure the continued success of TCAR. However, TCAR is not available in China. To the knowledge of the authors, there have been no published studies that determine the proportion of Chinese patients who meet the anatomic requirements of TCAR. The purpose of our study is to discover and investigate the anatomic characteristics of Chinese patients for TCAR.

## Materials and methods

### Data set and patient cohort

This single-center and retrospective study was approved by our Institutional Review Board (WZ-07) where patients with carotid artery stenosis (50–99%) were consecutively identified and analyzed. Carotid stenosis and morphology of all enrolled patients were determined by computed tomography angiography (CTA), and then carotid revascularization (CEA or TF-CAS) was performed. The degree of stenosis was calculated using the North American Symptomatic Carotid Endarterectomy Trial (NASCET) criteria (12). The criteria for revascularization were asymptomatic stenosis ≥70% or symptomatic stenosis ≥50%. For symptomatic carotid stenosis, the eligibility of patients was ipsilateral monocular blindness, transient ischemic attack (TIA), or stroke 180 days before carotid revascularization. Demographics and comorbidities of patients were reviewed by independent physicians and recorded.

### Eligibility for TCAR and CTA evaluation

First of all, similar to TF-CAS, patients who were eligible for TCAR should at least be suitable for stent implantation. Based on the instructions for the use of the Silk Road ENROUTE Transcarotid Neuroprotection System (shown in Supplementary Table 1), contraindications for the TCAR procedure include several anatomic features, such as the distal internal carotid artery (ICA) string sign (ICA < 4 mm), dilated or aneurysmal ICA with a diameter larger than the largest carotid stent (>9 mm), CCA diameter of < 6 mm, clavicle to carotid bifurcation distance of < 5 cm, and significant plaque at the intended CCA puncture site (8). Also, a CCA depth of >4 cm was considered more challenging. Although this anatomic characteristic was not strictly mentioned in contraindications, it would possibly induce higher risk during exposure for CCA access in patients with deeper necks (10). CTA studies were performed as per our institutional protocol using 2 mm slices, and three-dimensional reconstructions were subsequently created. Specifically, the following data were collected from CTA images: aortic arch type I, II, III, or bovine; aortic arch calcification grades; the diameters of CCA and ICA at bifurcation and skull base; CCA puncture site plaque and CCA ostium plaque grades; location of lesion calcification, graded as none, anterior, posterior, or circumferential; clavicle to carotid bifurcation distance; and CCA depth from the skin at the site of potential TCAR sheath access. Similar to Faneli's study (13), calcification grades were evaluated subjectively using CT scans. Arterial calcification patterns were examined semi-quantitatively: grade 1 (0–90°), grade 2 (0–180°), grade 3 (0–270°), and grade 4 (0–360°). The specific carotid measurements of TCAR in our study, using internal program tools, are shown in Supplementary Figure 1.

### Carotid revascularization

All CEA procedures were performed under general anesthesia, and the specific surgical methods can be referred to in our previous studies (14). In our center, shunts were routinely used for patients combined with contralateral carotid stenosis or lower back pressure (< 40 mmHg). Patch angioplasty was also routinely applied to close the arteriotomy for better reconstruction of the carotid bulb. As described in our previous research, all TF-CAS procedures were performed under local anesthesia by very experienced vascular surgeons in our institution. An embolic protection device (EPD; FilterWire EZ, Boston Scientific, Natick, MA) was routinely used in all cases before angioplasty and stent deployment. Pre-dilation was performed for lesions, and suitable stents were selected for implantation afterwards. Postoperative blood pressure (BP) was monitored by bedside monitors (BSM-2301, Nihon Kohden Corp., Japan) in the first 24 h. The hemodynamic instability was recorded and analyzed similarly to our previous study (15).

### Circle of Willis

The definition of the circle of Willis (CoW) was consistent with previous studies (16–18). We performed preoperative CTA of both extracranial and intracranial arteries. The images were further evaluated on a dedicated CT workstation (IntelliSpace Portal; Philips Healthcare). Normal CoW was constitutive of the anterior communicating artery (AcoA), both sides of the first segment of the anterior cerebral arteries (A1), the posterior communicating arteries (PcoA), the first segment of the posterior cerebral arteries (PCAs), and the basilar artery (BA). We defined the anterior semicircle of the CoW as the contralateral A1 segment to the ipsilateral middle cerebral artery (MCA; contralateral A1, AcoA, and ipsilateral A1). Likewise, we defined the ipsilateral posterior semicircle from the BA to the ipsilateral MCA (the ipsilateral first segment of the PCA and ipsilateral PcoA). The semicircle was incomplete if at least one segment was missing. The CoW morphology for each patient was assessed by two experienced radiologists. In consideration of the hemodynamic balance, we defined either the anterior semicircle of the CoW or the ipsilateral posterior semicircle as complete CoW. The CoW was incomplete if neither the anterior nor the posterior semicircle was visible from CTA images.

### High-risk anatomical characteristics of CEA and TF-CAS and peri-procedural complications

Similar to the previous study, high-risk anatomical characteristics for CEA include a hostile neck and high bifurcation extending behind the jaw. A hostile neck is defined as the patient having a prior history of neck radiation or neck surgery. Meanwhile, high-risk anatomical characteristics for TF-CAS include the absence of severe distal ICA tortuosity, type III aortic arch, and severe arch calcification (11). The peri-procedural complications of carotid revascularization in the present study include vasospasm, cranial nerve injury (CNI), and cervical hematoma. The clinical diagnosis of CNI and a description of the nerve(s) involved were detailed for every patient undergoing CEA. All surgeons examined patients for motor deficits involving the 7, 10, 11, and 12th cranial nerves as well as for the sympathetic chain (Horner syndrome) (19). Vasospasm usually results from “irritation” of the vessels by catheter manipulation during TF-CAS and is identified by interventionalists using angiography.

### Statistical analysis

All analyses were performed using SPSS V.22 software (IBM, Chicago, Illinois, USA). Frequency and percentage (%) were applied for categorical variables, which were then analyzed by χ^2^ test. Continuous variables that were analyzed by Mann–Whitney *U*-test were presented as mean ± standard deviation. Fisher exact test and two-sample *t*-test were also used for further comparison. Forward logistic regression (*P* < 0.1 for selection) was applied to identify the factors that were independently associated with anatomic ineligibility for TCAR. The significance level was set at *p* < 0.05 for all statistical tests.

## Results

### Overall cohort and TCAR eligibility

**Figure 1:**
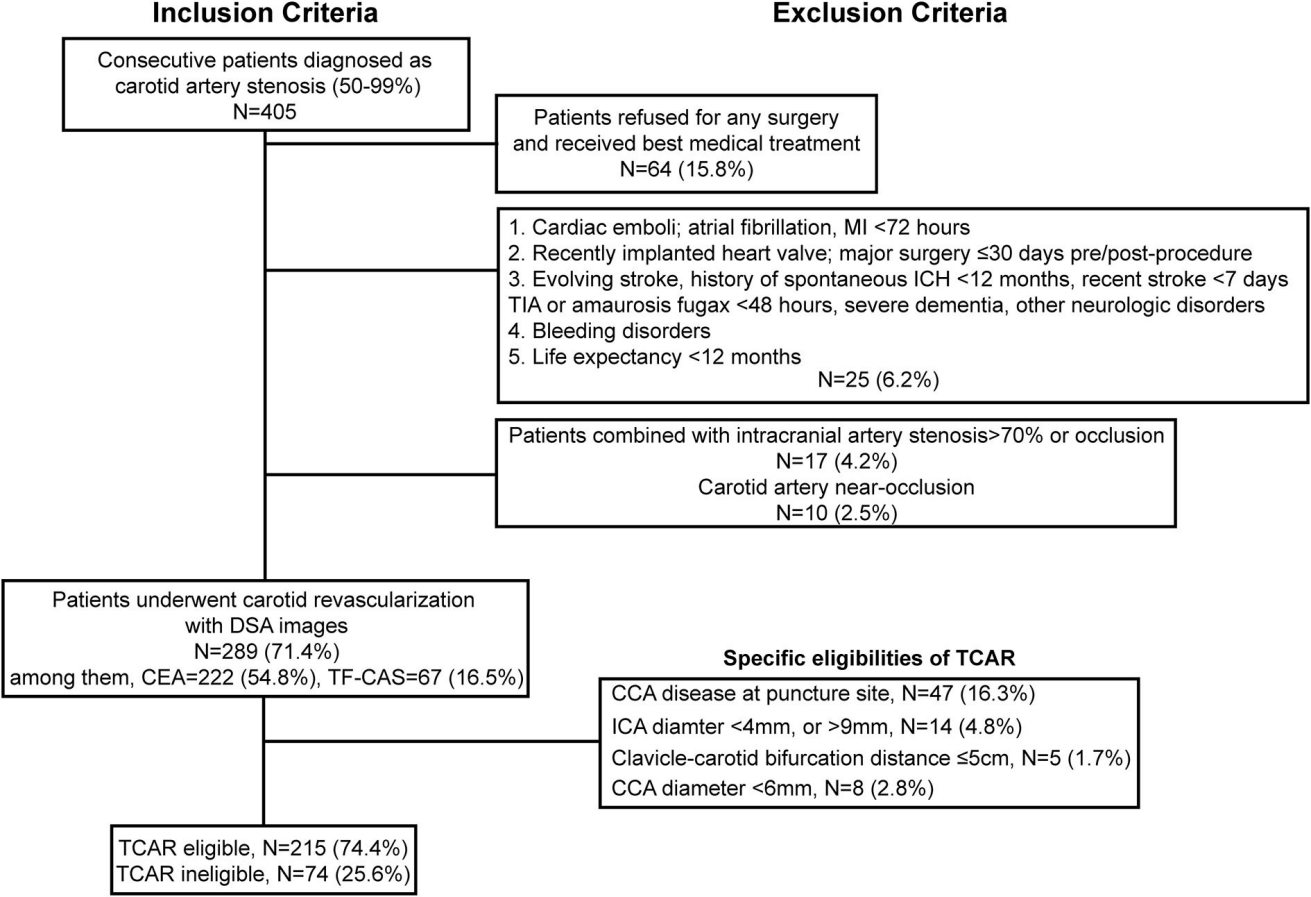
Inclusion and exclusion criteria of TCAR eligibility for patients with carotid stenosis who underwent carotid revascularization. MI, myocardial infarction; ICH, intracranial cerebral hemorrhage; TIA, transient ischemic attack; DSA, Digital substraction angiography; CEA, Carotid endarterectomy; TF-CAS, Transfemoral-Carotid artery stenting; CCA, common carotid artery; ICA, internal carotid artery; TCAR, transcarotid artery revascularization.

The flowchart of enrollment is shown in Figure 1, and a total of 289 patients who had undergone carotid revascularization including CEA and TF-CAS at our center from January 2019 to January 2021 were identified and enrolled. During the same period, a total of 222 CEA and 67 TF-CAS procedures were carried out. According to TCAR contraindications, a total of 215 patients remained for anatomic eligibility analysis. Specifically, a CCA diameter of >6 mm was noted in 277 arteries (95.8%), while an ICA diameter of 4–9 mm was found in 275 arteries (95.2%). The mean ICA diameter at the skull base was 4.7 ± 1.5 mm, and the mean ICA diameter at bifurcation was 5.8 ± 1.4 mm. Furthermore, 284 patients (98.3%) satisfied the required >5 cm clavicle–carotid bifurcation distance, 242 patients (83.7%) had minimal or no CCA puncture site plaques (grade 1 or 2), and no circular calcification was observed in 271 patients (93.8%).

### Demographics and comorbidities

Demographics and comorbidities for the cohorts were collected and are shown in Table 1. Patients who were ineligible for TCAR were more likely to be older (69.3 ± 8.8 vs. 77.5 ± 6.9y; *P* < 0.01) and female (40.5 vs. 16.3%; *P* = 0.01). In addition, patients who failed TCAR eligibility exhibited significantly higher body mass index (BMI; 26.0 ± 2.9 vs. 23.9 ± 2.7; *P* = 0.02) and nicotine abuse (54.1 vs. 40.5%; *P* = 0.04). Specifically, asymptomatic carotid stenosis was observed in 108 patients (37.4%), and severe carotid stenosis (70–90%) was found in the majority of the enrolled patients (204/289, 70.6%). No statistical significance was observed in comorbidities or carotid intervention history between the two groups.

**Table 1:** Baseline characteristics of patients who were eligible or not eligible for TCAR.

**Characteristics**	**Overall (*n* = 289)**	**TCAR eligible (*n* = 215)**	**TCAR ineligible (*n* = 74)**	***P*-value**
Age, years	69.7 ± 9.2y	67.0 ± 8.3y	77.5 ± 6.9y	< 0.01*
Female	65 (22.5%)	35 (16.3%)	30 (40.5%)	0.01*
BMI	24.4 ± 2.8	23.9 ± 2.7	26.0 ± 2.9	0.02*
Hypertension	207 (71.6%)	152 (70.7%)	55 (74.3%)	0.55
DM	104 (36.0%)	80 (37.2%)	24 (32.4%)	0.46
CAD	53 (18.3%)	40 (18.6%)	13 (17.6%)	0.84
Hyperlipidemia	219 (75.8%)	164 (76.3%)	55 (74.3%)	0.74
COPD	48 (16.6%)	34 (15.8%)	14 (18.9%)	0.54
CKD	32 (11.1%)	20 (9.3%)	12 (16.2%)	0.10
Smoker	127 (43.9%)	87 (40.5%)	40 (54.1%)	**0.04** ^*^
Asymptomatic	108 (37.4%)	80 (37.2%)	28 (37.8%)	0.92
Left side	152 (52.6%)	108 (50.2%)	44 (59.5%)	0.18
**Degree of CS**				
50–70%	85 (29.4%)	65 (30.2%)	20 (27.0%)	0.60
>70%	204 (70.6%)	150 (69.8%)	54 (73.0%)	
Prior contralateral intervention history	23 (8.0%)	18 (8.4%)	5 (6.8%)	0.66

TCAR, transcarotid artery revascularization; BMI, body mass index; DM, diabetes, mellitus; CAD, coronary artery disease; COPD, chronic occlusive pulmonary disease; CKD, chronic kidney disease; CS, carotid stenosis.

Continuous data are presented as means ± SD.

^*^P < 0.05.

### Arterial characteristics

The carotid and aortic anatomy of all patients enrolled were categorized, and the results are shown in Table 2. Notably, mild CCA ostium plaque (grades 1 and 2) was mostly found in both TCAR eligible or ineligible groups (196, 91.2% vs. 67, 90.5%). Although not strictly required on contraindications, an extended CCA depth was characterized to be more technically challenging for TCAR. In our cohort, 65 patients (22.5%) were found to have a CCA depth exceeding 4.0 cm. For TCAR contraindications, anatomic characteristics of the aorta arch were also assessed. In general, 95 of them had type III aortic arch (32.9%) and 30 of them had bovine arch (10.4%). A total of 111 patients (38.5%) had severe arch calcification (grades 3 and 4). The CoW has already been testified for its key role in ischemia protection. The presence of hemodynamic compensation using CoW may be clinically beneficial for defining the risk of intra-operative ischemia in patients with carotid stenosis. During the TCAR procedure, the configuration of CoW seems to be significant to establish temporary dynamic flow reversal. As shown in Table 2, incomplete CoW (neither AcoA nor PcoA) was noted in 96 patients (33.2%), while complete CoW was found in a total of 193 patients (66.8%). Notably, 71 patients with TCAR eligibility (33.0%) were shown to be combined with incomplete CoW.

**Table 2:** Aortic arch and carotid-intracranial artery characteristics of patients who were eligible and ineligible for TCAR.

**Artery characteristics**	**Patients enrolled (*n* = 289)**	**TCAR eligible (*n* = 215)**	**TCAR ineligible (*n* = 74)**	***P*-value**
**CCA ostium plaque**				
Grade 1	239 (82.7%)	178 (82.8%)	61 (82.4%)	0.47
Grade 2	24 (8.3%)	18 (8.4%)	6 (8.1%)	
Grade 3	14 (4.8%)	12 (5.6%)	2 (2.7%)	
Grade 4	12 (4.2%)	7 (3.3%)	5 (6.8%)	
**CCA depth**				
< 4.0 cm	224 (77.5%)	170 (79.1%)	54 (73.0%)	0.29
4.0–4.5 cm	36 (12.5%)	27 (12.6%)	9 (12.1%)	
>4.5 cm	29 (10.0%)	18 (8.4%)	11 (14.9%)	
**Aortic arch type**				
Type I	91 (31.5%)	65 (30.2%)	26 (35.1%)	0.41
Type II	73 (25.3%)	64 (29.8%)	9 (12.2%)	
Type III	95 (32.9%)	68 (31.6%)	27 (36.5%)	
Bovine arch	30 (10.4%)	18 (8.4%)	12 (16.2%)	
**Aortic arch calcification**				
Grade 1	64 (22.1%)	48 (22.3%)	16 (21.6%)	0.39
Grade 2	114 (39.4%)	51 (23.7%)	11 (14.9%)	
Grade 3	62 (21.5%)	34 (15.8%)	15 (20.3%)	
Grade 4	49 (17.0%)	82 (38.1%)	32 (43.2%)	
**Completeness of CoW**				
AcoA+PcoA	41 (14.2%)	33 (15.3%)	8 (10.8%)	0.15
AcoA alone	108 (37.4%)	84 (39.1%)	24 (32.4%)	
PcoA alone	44 (15.2%)	27 (12.6%)	17 (23.0%)	
None	96 (33.2%)	71 (33.0%)	25 (33.8%)	
Complete CoW	193 (66.8%)	144 (67.0%)	49 (66.2%)	0.91
Incomplete CoW	96 (33.2%)	71 (33.0%)	25 (33.8%)	

TCAR, transcarotid artery revascularization; CCA, common carotid artery; CoW, circle of Willis; AcoA, anterior communicating artery; PcoA, posterior communicating artery.

Continuous data are presented as means ± SD.

### Binary logistic regression for TCAR ineligibility

As shown in Table 3, the binary logistic regression model was adopted for identifying factors independently associated with TCAR ineligibility. Our model involved all variables (*P* < 0.1). Female patients were discovered to be associated with TCAR ineligibility (OR, 5.967; 95% CI: 2.545–13.987; *P* < 0.001). Similarly, older age (OR, 1.226; 95% CI: 1.157–1.299; *P* < 0.001) and higher BMI (OR, 1.462; 95% CI: 1.260–1.697; *P* < 0.001) were independently associated with TCAR ineligibility. Other variables illustrated non-significance in this regression model (*P* > 0.05).

**Table 3:** Factors independently associated with TCAR ineligibility.

**Characteristics**	**OR**	**95% CI**	***P*-value**
Female	5.967	2.545–13.987	**< 0.001** ^*^
Age	1.226	1.157–1.299	**< 0.001** ^*^
BMI	1.462	1.260–1.697	**< 0.001** ^*^
Smoking	0.973	0.468–2.024	0.941
CKD	2.099	0.658–6.689	0.210
Left side	0.955	0.464–1.967	0.901

TCAR, transcarotid artery revascularization; OR, odds ratio; CI, Confidence interval; BMI, body mass index; CKD, chronic kidney disease.

^*^P < 0.05.

### Patient characteristics of CEA and TF-CAS toward TCAR eligibility

All patients who had carotid revascularization by either CEA or TF-CAS were divided into two groups in accordance with TCAR eligibility. Demographics, lesion characteristics, and peri-procedural complications are shown in Table 4. Female patients and those of an older age were also statistically different with regard to TCAR eligibility (*P* < 0.01). Non-significance was found in other demographic variables. High risk for CEA was observed in 29 patients (17.3%) with TCAR eligibility and six patients (11.1%) without TCAR eligibility. Meanwhile, a high risk for TF-CAS was observed in nine patients (19.1%) with TCAR eligibility and six patients (30.0%) without TCAR eligibility. Generally, CNI was found in 22 patients undergoing CEA, while 19 of them (11.3%) met the TCAR eligibility. Moreover, cervical hematoma, a common complication after CEA, was noticed in 10 patients, and vasospasm was observed in 11 patients receiving TF-CAS. Non-significance was visible between TCAR eligibility groups (*P* > 0.05).

**Table 4:** Patient characteristics of CEA and TF-CAS, eligible vs. ineligible for TCAR.

**CEA**, ***n*** = **222**				**TF-CAS**, ***n*** = **67**		
**TCAR eligibility**	**Eligible**,	**Ineligible**,	***P*** **-value**	**Eligible**,	**Ineligible**,	***P*** **-value**
	***n*** = **168**	***n*** = **54**		***n*** = **47**	***n*** = **20**	
Age, y	67.1 ± 8.8	78.1 ± 6.7	**< 0.01** ^*^	66.4 ± 6.2	75.9 ± 7.3	**< 0.01** ^*^
Female	29 (17.3%)	19 (35.2%)	**< 0.01** ^*^	6 (12.8%)	11 (55.0%)	**< 0.01** ^*^
Smoking	66 (39.3%)	21 (38.9%)	0.96	21 (44.7%)	8 (40.0%)	0.72
HTP	120 (71.4%)	42 (77.8%)	0.39	32 (68.1%)	13 (65.0%)	0.99
Hyperlipidemia	127 (75.6%)	38 (70.4%)	0.48	37 (78.7%)	17 (85.0%)	0.74
DM	65 (38.7%)	13 (24.1%)	0.07	15 (31.9%)	11 (55.0%)	0.10
CAD	29 (17.3%)	10 (18.5%)	0.83	11 (23.4%)	3 (15.0%)	0.66
Left side	78 (46.4%)	31 (57.4%)	0.21	30 (63.8%)	13 (65.0%)	0.99
High-risk for intervention	29 (17.3%)	6 (11.1%)	0.28	9 (19.1%)	6 (30.0%)	0.51
**Peri-procedural complications**						
CNI	19 (11.3%)	3 (5.6%)	0.22	0	0	-
Cervical hematoma	8 (4.8%)	2 (3.7%)	0.99	0	0	-
Vasospasm	0	0	-	8 (17.0%)	3 (15.0%)	0.99

CEA, carotid endarterectomy; TF-CAS, transfemoral carotid artery stenting; TCAR, transcarotid artery revascularization; HTP, hypertension; DM, diabetes mellitus; CAD, coronary artery disease; CNI, cranial nerve injury.

Continuous data are presented as means ± SD.

^*^P < 0.05.

## Discussion

Each of these procedures has certain anatomic constraints that may affect the outcome, or make one approach ideal

while another is contraindicated. Thus, previous studies were conducted on anatomic criteria for TCAR, CEA, and TF-CAS in patients undergoing carotid revascularization, and comparisons were made between eligible and ineligible groups. According to Wu et al.'s study, ~68% of arteries were eligible for TCAR and 76% were eligible for TF-CAS, while 85.0 and 64.9% were found to be eligible in Kumins et al.'s study, respectively (10, 11). These results are somewhat similar to ours showing that 215 (74.4%) of 289 carotid arteries were considered eligible for TCAR. In this study, the Chinese patients enrolled had a relatively longer distance from clavicle to carotid bifurcation (83.7 ± 12.4 mm), whereas a decreased distance was found in both Wu's and Kumins' studies [6.1 (5.1–7.1) cm and 72.4 ± 13.5 mm]. The distance from the clavicle to carotid bifurcation is vital and fairly inviolate because the sheath extends 2 cm into the CCA, and it is mounted on an inner dilator that protrudes 4 cm from the entry site (10).

Previous studies have identified the potential demographic and comorbidity characteristics that may predispose individuals to anatomic variants compatible or incompatible with TCAR (20). Carotid arteries of American patients that were anatomically ineligible for TCAR were more probably associated with age, hyperlipidemia, COPD, and calcification thickness. In the study conducted by Wu et al., older age was independently associated with TCAR ineligibility. Older patients were prone to the development of thoracic kyphosis, which might subsequently compress anterolateral neck structures and lower the clavicle–carotid bifurcation distance necessary for TCAR (21). Similarly, we identified such associations in our cohort. To be specific, female patients, being of an older age, and having a higher BMI were each significantly associated with TCAR ineligibility. In our cohort of Chinese patients, the mean BMI was 23.9 ± 2.7 in the TCAR eligible cohort, while that in Wu et al.'s study was remarkably higher (28.3 ± 5.5 in the TCAR eligible patients), suggesting that American patients were more likely to have problems of obesity. Indeed, patients with obesity may be highly associated with having a CCA depth of >4 cm due to the thickness of subcutaneous fat, and increased depths are identified as being related to increased difficulty in both exposure and TCAR system insertion. Meanwhile, Chinese female patients in our cohort were more likely to have an ICA diameter of < 4 mm, making carotid arteries anatomically ineligible for TCAR. This result may be attributable to the chronic damage to carotid arteries resulting from inflammatory disease, such as Takayasu's disease, which disproportionately affects young females of Asian descent (22, 23).

The circle of Willis is normally seen at the AcoA and PcoA openings. In our study, we further explored the completeness of the CoW using CT angiography. Although a complete and fully functional CoW exists only in ~30% of elderly (arteriosclerotic) patients, more studies have reached a consensus that collateral insufficiency impairs cerebrovascular reactivity (CVR), elongates cerebral circulation time (CCT), and aggravates the risk of subsequent ischemic events as well as border zone infarcts (24–26). Our previous study showed that complete CoW lowers the incidence of immediate neurologic events (INEs) in patients with severe carotid stenosis after CEA (18). Although the CoW opening cannot be seen in cases of hemodynamic balance and was not included in contraindications of TCAR, AcoA, or PcoA were shown to be the critical pathways during the TCAR procedure, with occlusion or hypoplasia of them resulting in insufficient blood flow to ipsilateral hemicerebrum and ultimately watershed infarction (24, 26). In this study, among 215 patients with TCAR eligibility, incomplete CoW was found in 71 patients (33.0%), indicating that presumable higher cerebral ischemia risk would be increased during the TCAR procedure. In general, no comparable studies have been made to explore the role of CoW in the TCAR procedure. Therefore, the configuration of CoW should be taken into consideration in these high-risk patients.

Recent findings demonstrate that patients undergoing TCAR experience reduced operative times, lengths of stay, and cranial nerve injuries (20). When working with a hostile neck during CEA, it is increasingly challenging to identify a defined endarterectomy endpoint, and higher rates of CNI may be induced (27–29). Our results were consistent with these findings showing that a total of 19 patients (11.3%) encountered CNI after CEA, and all of them met TCAR eligibility. As a result, the incidence of CNI would be greatly reduced in high-risk patients who were eligible for TCAR because there would be no need for adequate exposure to ICA, and symptoms such as dysphagia, loss of gag reflex, and aspiration pneumonia might not occur. Similar findings were seen in the occurrence of vasospasm, which was significantly higher in patients who underwent the TF-CAS procedure. In general, the elevated risk of vessel disorder and intra-operative distal embolization is associated with TF-CAS while manipulating a diseased aortic arch, crossing the carotid lesion with the Guidewire, and crossing the lesion with the neuroprotection filter device itself (30).

However, this study has several limitations. First, the calcification of carotid arteries assessed in our cohort was subjectively measured based on a grading scale. In addition, the clavicle–carotid bifurcation distances assessed by CTA in our study could have been underestimated because the threshold of working length (≥5 cm) was determined by the ENROUTE IFU ultrasound. Furthermore, the anatomical basis of CoW already exists, but it is closed temporarily, and its functional activity depends on demand. Hence, it is hard to analyze the completeness of CoW in all patients with carotid stenosis simply from observation. Finally, more focused research involving different nations and races is required to further define and identify the anatomical characteristics that are related to favorable or adverse outcomes after TCAR so that it may serve as a feasible and alternative carotid revascularization strategy for carotid artery disease.

## Conclusion

Transcarotid artery revascularization is a readily available treatment option for many patients with carotid stenosis, with irreplaceable merits and limited anatomic requirements. While there are differences in TCAR practice between nations and races, this study showed that the majority of Chinese patients enrolled in this study were suitable for TCAR, while female patients, being of older age, and having a higher BMI were each identified as independent factors for TCAR eligibility. Although not strictly contained in IFUs, the configuration of CoW should be taken into consideration when performing TCAR. Finally, by a complete analysis of anatomic features with larger samples, TCAR could become a favorable surgical technique for the management of carotid artery stenosis in Chinese patients, and a vascular surgeon should be able to provide all revascularization options for favorable determination.

## Data availability statement

The raw data supporting the conclusions of this article will be made available by the authors, without undue reservation.

## Ethics statement

Written informed consent was obtained from the individual(s) for the publication of any potentially identifiable images or data included in this article.

## Author contributions

WF, WS, and BY: conception and design. WG and JTa: analysis and interpretation. WG and JTo: data collection. WF, SL, and WS: writing the article. WF, WS, SL, JTa, and BY: critical revision of the article. WF, WS, SL, WG, JTo, JTa, and BY: final approval of the article. WF: statistical analysis. BY: overall responsibility. All authors contributed to the article and approved the submitted version.
