# Supplementary material for: Analysis of the anatomic eligibility for transcarotid artery revascularization in Chinese patients who underwent carotid endarterectomy and transfemoral carotid artery stenting
Source: Front Cardiovasc Med. 2023 Jan 6;9:1045598. doi: 10.3389/fcvm.2022.1045598 (PMC9852860; doi:10.3389/fcvm.2022.1045598)
Supplement: Supplementary file 1 [file Table_1.docx]

**Supplementary table 1.** eligibility criteria of TCAR

| **Specific eligibilities** | **General eligibilities (similar to TF-CAS)** |
| --- | --- |
| No CCA disease at puncture site | No cardiac emboli and atrial fibrillation; no MI within 72 hours |
| clavicle to bifurcation distance >5cm | No recently implanted heart valve; No major surgery ≤30 days |
| CCA diameter >6mm | No history of spontaneous ICH <12 months, No recent stroke <7 days, |
| ICA diameter 4-9mm | No bleeding disorders |
| No contralateral lateral recurrent, laryngeal, or vagus nerve injury | Life expectancy >12 months |
|  | No previous stent in target vessel; No CCA/ICA occlusion or string sign |
|  | Without severe ostial lesion |
|  | Without ipsilateral intracranial or extracranial stenosis |
|  | No contraindication to aspirin, clopidogrel or anticoagulation |

Notes: TCAR, transcarotid artery revascularization; TF-CAS, transfemoral carotid artery stenting; CCA, common carotid artery; ICH, intracranial hemorrhage; ICA, internal carotid artery;
