# Supplementary figures and images for: Analysis of the anatomic eligibility for transcarotid artery revascularization in Chinese patients who underwent carotid endarterectomy and transfemoral carotid artery stenting
Source: Front Cardiovasc Med. 2023 Jan 6;9:1045598. doi: 10.3389/fcvm.2022.1045598 (PMC9852860; doi:10.3389/fcvm.2022.1045598)

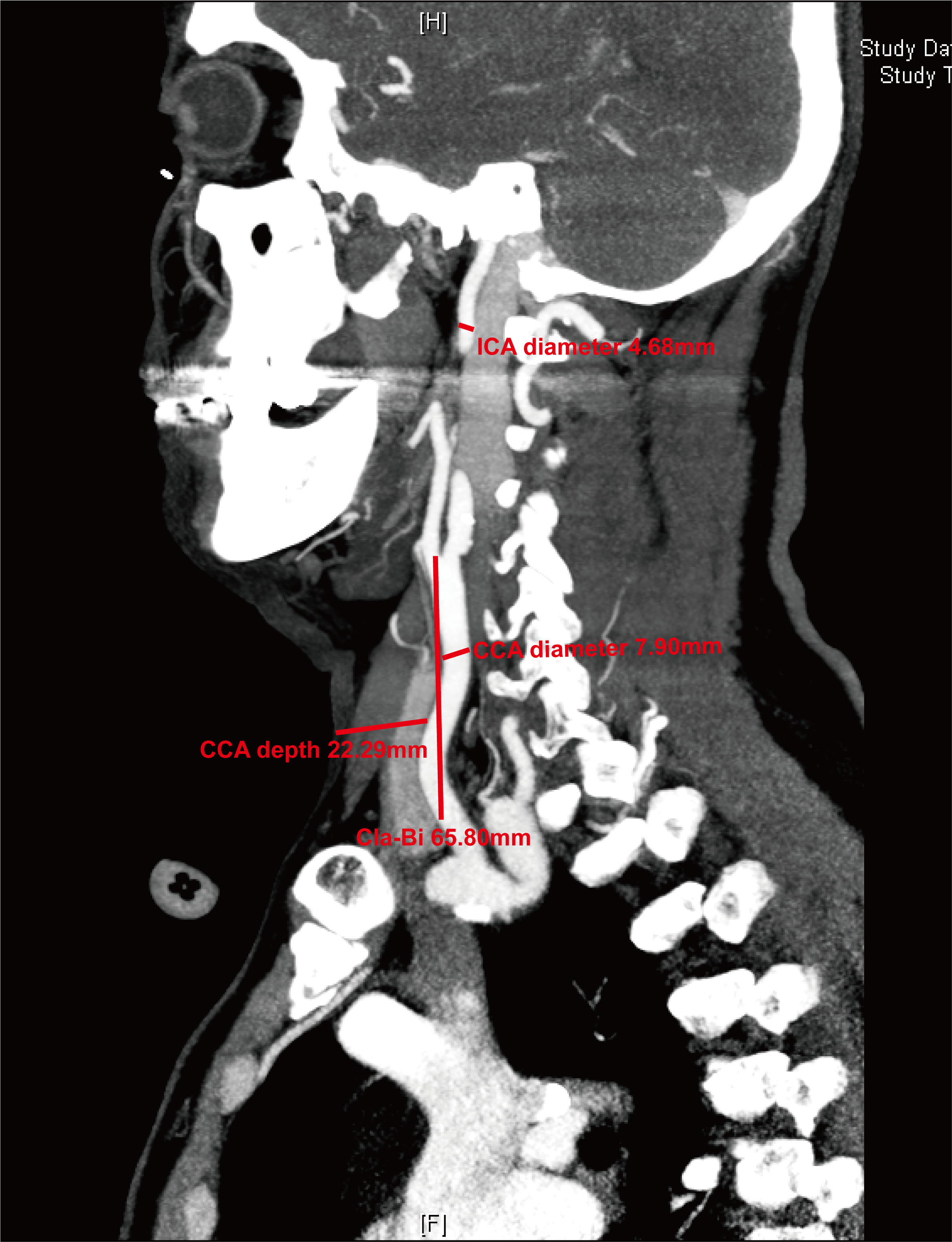

Supplement: Supplementary file 2 [file Image_1.TIF]
